# Clinical Analysis of Improved Particle Swarm Algorithm-Based Magnetic Resonance Imaging Diagnosis of Placenta Accreta

**DOI:** 10.1155/2021/7373637

**Published:** 2021-08-13

**Authors:** Xiaoyan Ding, Yingying Cao, Fengtao Sun, Airong Ma, Feiyue Zhang

**Affiliations:** Department of Obstetrics, Zibo Central Hospital, Zibo 255036, Shandong, China

## Abstract

The magnetic resonance imaging (MRI) image processing capabilities were investigated based on the improved particle swarm optimization (IPSO) algorithm, and the clinical application analysis of MRI images in the diagnosis of placenta accreta (PA) was evaluated in this study. The MRI uterine images were detected on the basis of IPSO. Besides, the clinical data of 89 patients with PA were selected and collected, who were diagnosed by clinical cesarean section surgery and pathological comprehensive diagnosis in hospital from January 2018 to July 2020. Then, all of them underwent the ultrasound (US) and MRI examinations, and the differences of sensitivity, specificity, and accuracy between MRI and US under IPSO in the diagnosis of PA were compared, as well as the differences in the diagnosis of adhesive, implantable, and penetrated PA. The results showed that the difference in detection between IPSO-based MRI images and US images was not statistically substantial (*p* > 0.05), but the number of initial detections was higher than the number of US examination. MRI examination had higher sensitivity and specificity in the diagnosis of PA during pregnancy, especially for implantable PA, compared with US examination (*p* < 0.05). In conclusion, MRI images based on the improved particle swarm optimization algorithm showed a good application effect in the diagnosis of placental implantation diseases, which was worthy of further promotion in clinical practice.

## 1. Introduction

Placenta accreta (PA) means that the decidua between the placenta and the uterus has not been fully developed or has traumatic defects, causing the placenta to directly adhere to the myometrium or even directly penetrate the myometrium [1]. With the increasing risk factors of PA, the incidence rate is increasing year by year. Pathologically, PA is divided into three grades based on the depth of placental villi implantation into the myometrium, including adhesive, implantable, and penetrated PA. As a serious complication of pregnancy, it can lead to maternal postpartum hemorrhage and death [2]. For this reason, early diagnosis and detection of PA and targeted treatment are of positive significance for improving prognosis. Early PA has no typical symptoms or only mild abdominal pain and vaginal bleeding, which are easily ignored or misdiagnosed as other diseases [3].

With the continuous advancement of science and technology, medical imaging methods have also made considerable developments. At present, the more common forms of medical imaging include image diagnosis, medical ultrasound, X-ray tomography, and magnetic resonance imaging (MRI). As a new and rapidly developing examination method, MRI is an examination that uses the phenomenon of magnetic resonance to generate magnetic resonance signals to form images. Compared with computed tomography (CT), digital subtraction angiography (DSA), Doppler tissue imaging (DTI), and other imaging techniques, it has the advantages of high spatial resolution, high soft tissue contrast, multiple imaging parameters, large amount of information, noninvasiveness, and low damage, so MRI can also be used for examination of pregnant women. It has been extensively used in medical institutions and scientific research institutes at all levels as well as enterprise research and development institutions and has become one of the commonly applied research methods in the field of disease diagnosis and treatment neuroscience research and experiment [4]. At present, ultrasound (US) and MRI are frequently used imaging methods in the clinical diagnosis of prenatal PA [5]. Among them, US has the highest application rate, but US examination is easily affected by many factors, such as lipid, placental position, and amniotic fluid, which reduce the accuracy of the examination. Due to its good contrast resolution, multidirectional and multiparameter imaging, and flexible imaging position [6], MRI can show the status of the placenta of pregnant women more clearly and provide a more comprehensive and detailed basis for clinical diagnosis and surgery. Therefore, it is gradually applied to the diagnosis of PA. The role of MRI in diagnosing PA and surgical decision-making has also been paid more and more attention by physicians. As a noninvasive and nonionizing radiation examination, the wide application of MRI provides a lot of accurate data support for the classification of PA.

The simplified social model of birds is originated by PSO. During its search iteration process, the particles constantly adjust their respective directions through mutual communication and learning, but no matter how scattered they are, they eventually gather in one direction. In this process, the information sharing mechanism between groups is employed to exchange information, and the optimal solution is found through individual cooperation [7]. Since it has been proposed, many researchers have paid attention to the improvement and optimization of PSO and applied it to practical problems due to its intelligent idea, simple and easy implementation process, and fast search speed of examples [8].

The content of this study was to analyze the value of MRI images based on IPSO in diagnosing PA compared with US examination, hoping to provide some help in the detection and treatment of PA.

## 2. Materials and Methods

### 2.1. Research Objects and Grouping

The clinical data of 89 patients with PA diagnosed by clinical cesarean section surgery and pathological comprehensive diagnosis in hospital from January 2018 to July 2020 were collected for this study. The age range of pregnant women was 24–45 years old, with an average of 37.04 years. All gestational weeks were determined by US, and the gestational age was 15–39 weeks (average 32.92 weeks) after MRI. The main symptom of the patient was irregular vaginal bleeding. The process had been approved by the ethics committee of hospital, and all the research objects included in this study had signed the agreement.

The inclusion for criteria were defined to include patients who had complete preoperative clinical history data, were greater than 18 years old, were in the middle and late pregnancy, had complete imaging data before surgery (all patients at the same gestational age underwent US and MRI examinations) and received treatment and delivery, with complete surgical records and pathological results, and carried out the all involved examinations, clinical treatments, and theoretical analysis within the scope of ethics, with the informed consent of each patient and her family members.

The exclusion for criteria were defined to include patients who had the image quality that did not meet the standard and affect the observation, were combined with other severely harmful complications during pregnancy, endangering the life of the pregnant woman and the fetus, suffered from major diseases, such as impaired heart, liver, and kidney function, blood system diseases, malignant tumors, and mental illnesses, which affected the growth and development of the fetus during pregnancy were restricted, and endangered the health and life of the fetus.

### 2.2. MRI Image Segmentation Algorithm Based on the Improved Particle Swarm Algorithm

**Figure 1:**
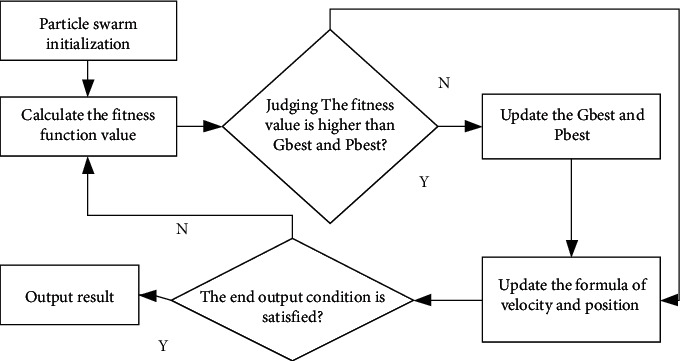
The basic process of PSO.

The basic flow of PSO is shown in Figure 1.

On the basis of following the design principles of PSO, PSO was improved by introducing a shrinkage factor to make the algorithm achieve effective calm between global search and local mining, in order to more effectively control the classification speed of particles and prevent particles from deviating from falling into the local optimum. Besides, the shrinkage factor *P* was set to a linear decreasing mode, the inertia weight was adopted to improve the speed of particle iteration, and the speed of particle iteration update was controlled in the most reasonable range, as shown in the following equation.

(1) $$\begin{array}{c} P=P_{\max}-\left(P_{\max}-P_{\min}\right)\cdot \left(\frac{n}{n_{\max}}\right). \end{array}$$

In the above equation, *P* represents the shrinkage factor, *P*_max_ and *P*_min_ stood for the upper bound of the shrinkage factor and the lower bound of the shrinkage factor, *n* represents the current iteration number, and *n*_max_ indicates the maximum iteration number. Compared with the inertial weight, the shrinkage factor could better constrain and control the flying speed of the particles and enhance the local search ability of the particles. In order to prevent the particles in PSO from converging to the local optimal solution, the particles should be distributed around the local optimal solution as much as possible because the global optimal solution was unknown in the search process. After a certain number of iterations, the position of the local optimal solution found by the particle swarm optimization algorithm was close to that before the global optimal solution [9].

In order to judge the performance of image segmentation algorithms, it was necessary to evaluate various segmentation algorithms. Segmentation evaluation was a critical means to promote the performance of the existing algorithm, optimize segmentation quality, and guide new algorithm. In this study, the following equation was adopted for quantitative calculation.

(2) $$\begin{array}{c} J\left(X_{1},X_{2}\right)=\frac{\left|X_{1}\cap X_{2}\right|}{\left|X_{1}\cup X_{2}\right|}. \end{array}$$

In the above equation, *X*_1_ represents the area of the standard sample in the database, and *X*_2_ represents the area of the region obtained by the algorithm segmentation in this study. The numerator was the common area of the two parts, and the denominator was the sum of the common area, the area of the original target area in the standard sample that was not divided, and the area of the area that was not in the standard sample that was erroneously divided. First, the MRI image was for the preprocessing operation, and the gray scale transformation was carried out to enhance the contrast between the target area and the background area in the image. Then, the image was for binarization operation to find the connected area in the image. According to the area of the connected area, some unrelated areas could be eliminated. Finally, IPSO combined with the maximum entropy algorithm was employed to segment the target area on the processed image.

**Figure 2:**
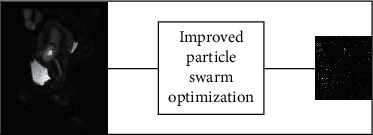
Detection process based on IPSO.

In this study, 89 patients' MRI images were selected, and IPSO-based segmentation algorithm and preprocessing algorithm were adopted to segment the placenta in the MRI image. The linear gray scale of the original MRI image was enhanced, the clarity of the target and the background was increased, and parameters were adjusted to find some connected areas in the image. The area was set as the background color, and finally, the boundary area was eliminated through Gaussian filtering. The target area was segmented by PSO, and the segmentation achieved good accuracy and resolution (Figure 2).

After the design of the improved particle swarm optimization algorithm for MRI images was completed, the algorithm was put into the HGG + LGG training set for testing, and the stability of MRI processing of the algorithm was tested by using the 50%-fold cross-validation method. The final parameter information was determined through continuous data debugging.

### 2.3. Research Methods

When US was performed, there was the transabdominal combined with transvaginal examination. Before the examination, pregnant women were routinely instructed to lie supine on the examination bed, and the patient could be scanned in a lateral position if necessary and the state of the pregnant woman allowed. The bladder should be moderately filled (the bladder can be empty on vaginal US), and then, the placenta should be further examined in full section. Before MRI scanning, the patient needed to hold the urine properly to fill the bladder to achieve a clear image of the bladder wall. The appropriate position was chosen according to the situation of the pregnant woman. For the patient with a high uterus, the breathing gated and nonapnea sequences were applied to relieve the mother discomfort, increase the degree of coordination, and do not use contrast enhancing agents.

Two experienced senior associate chief physicians with more than 5 years of experience would jointly perform blind reading of the medical imaging pictures obtained and try to avoid the participation of subjective factors that may affect the results. In case of disagreement between the 2 physicians, a third physician with the same senior experience shall further analyze and study the disputed image results, and the final result shall be subject to the consensus of the three physicians after consultation. The placental signal uniformity at the shape boundary of the placenta was observed and recorded.

### 2.4. Diagnostic Criteria

The results of surgical pathological examinations were used as the standard to evaluate the results of MRI diagnosis of patients, compare the sensitivity and specificity of the US and MRI examination methods in the diagnosis of PA, and analyze the consistency between the two groups and the intraoperative or pathological confirmation. The US group was then further compared with the MRI group in terms of the types of PA they diagnosed.

### 2.5. Statistical Methods

SPSS22.0 software was used for statistical analysis in this study, and the count data were expressed as the number of cases or percentages. Besides, the chi-square test was used for comparison (the sensitivity and specificity comparison adopted the paired chi-square test), and the measurement data were represented by mean ± standard deviation, with the *t*-test for comparison. The inspection level *α* = 0.05, and the difference was considered to be statistically substantial when *p* was less than 0.05.

## 3. Results

### 3.1. MRI Image Segmentation Based on the Improved Particle Swarm Algorithm

**Figure 3:**
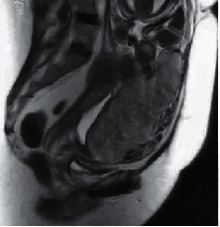
MRI image of adhesive PA.

**Figure 4:**
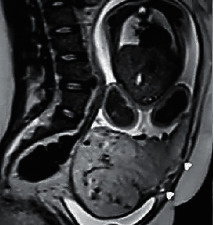
MRI image of implantable PA.

**Figure 5:**
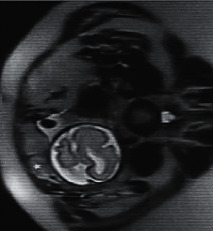
MRI image of penetrated PA.

Through the segmentation method based on IPSO to process MRI images, the following 3 typical images of PA could be obtained. Adhesive PA (Figure 3) showed that the villi adhered to the myometrium and could not dissect spontaneously. Implantable PA (Figure 4) revealed that the villi invaded the myometrium. For penetrated PA (Figure 5), the villi penetrated the serosal layer of the uterus.

### 3.2. Comparison on the Efficiency of MRI and US Examinations in Diagnosing PA

**Figure 6:**
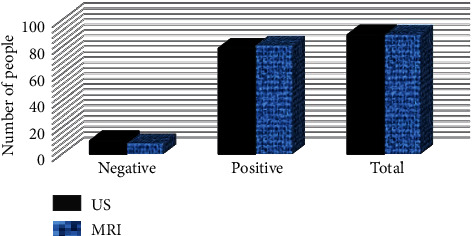
Comparison on MRI and US examination results with pathological examination results.

**Figure 7:**
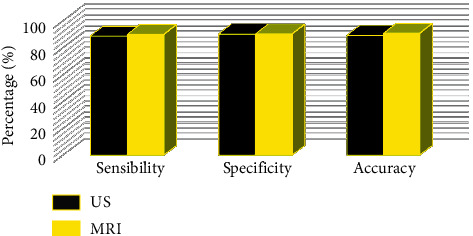
Comparison on the efficiency of US and MRI examinations in the diagnosis of PA.

The comparison results of US and MRI in diagnosing PA and pathological examination are shown in Figure 6. The results indicated that the number of cases confirmed by MRI and US examinations was 81 and 79, respectively; the number of cases confirmed by MRI examination was slightly higher than the number of cases confirmed by US examination, but the difference between the two results was not marked (*p* > 0.05). The comparison result in diagnosing PA is presented in Figure 7, suggesting that the sensitivity of MRI and US images after the improved particle swarm optimization was 88.76% and 90.01%, the accuracy was 89.36% and 90.78%, respectively, and the specificity was both 90.38%. Thus, there was no huge difference between the two groups of data (*p* > 0.05).

### 3.3. Comparison on MRI and US Examinations in Diagnosing the Types of PA

**Figure 8:**
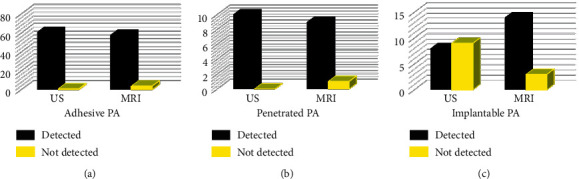
Comparison on US and MRI examinations in diagnosing the types of PA. (a) Adhesive PA. (b) Penetrated PA. (c) Implantable PA.

The results of MRI and US examinations in diagnosing the types of PA are shown in Figure 8. When diagnosing adhesive PA (Figure 8(a)) and penetrated PA (Figure 8(b)), there was no statistically obvious difference between MRI and US (*p* greater than 0.05). When diagnosing the implantable PA (Figure 8(c)), the difference was statistically marked (*p* less than 0.05), indicating that the diagnosis of implantable PA was implanted, and the effect of MRI was superior to US, which had a high diagnostic value.

## 4. Discussion

In this study, PSO was improved and applied to the MRI image. Based on the basic PSO, the parameters such as the shrinkage factor were optimized, the spatial position of the particles was adjusted, and the MRI image analysis of the particle swarm was improved. PA is a complication that seriously threatens the life and health of pregnant women. In recent years, with the increase of elderly women and the growth of cesarean section rate, the incidence of PA has been rising year by year [10]. The gold standard for diagnosis is that the placenta cannot be completely peeled off during delivery, the surface is not smooth, or the pathological results show abnormal structure of the placenta. The classification of PA can be divided into adhesive, implantable, and penetrated PA according to the different depths of placental villi invading into the myometrium [11]. Therefore, it is very important to accurately diagnose and evaluate the depth and location of PA before delivery.

At present, US is usually the preferred imaging examination technique when diagnosing PA [12] because the US operation is harmless and painless, simple, convenient, and flexible. Since the US instrument is small, it can be moved to any place. It is rare that patient examinations cannot be performed due to the patient's own reasons or hospital environment restrictions. However, US also has certain limitations. Its operation is susceptible to the subjective factors of the examining doctor. In addition, its low spatial resolution results in poor diagnostic efficiency of the posterior wall placenta, and it is also susceptible to the influence of amniotic fluid volume and the thickness of adipose tissue in the abdominal wall of pregnant women, which will have a certain impact on the diagnostic efficiency [13, 14]. Nowadays, with the continuous development and innovation of imaging technology, there are more and more applications of magnetic resonance in PA during pregnancy, and its diagnostic value has been affirmed. In the diagnosis of PA, MRI has dealt with the influence caused by the limitations of US to a certain extent. The imaging is relatively stable, which not only has a high diagnostic accuracy but also provides more information for the early development of clinical treatment plan. Meta-analysis studies have shown that MRI and US are equivalent in diagnosing PA [15], suggesting that MRI not only has a higher accuracy in the diagnosis of PA but also can show the changes in placenta morphology and tissue structure more clearly and intuitively. The latest metastudy has pointed out that MRI is very accurate in the diagnosis of PA [16], with a combined sensitivity of 94.4%, a combined specificity of 84.0%, and a diagnostic odds ratio of 89.0%. In recent years, MRI has a high accuracy in diagnosing the severity of prenatal PA and can observe other combined lesions, such as fetal brain development and intracranial hemorrhage. Therefore, MRI can be used as an important inspection method for PA during pregnancy [17].

In this study, after comparing the diagnostic efficacy of US and MRI examination in placenta implantation, it was found that the diagnostic efficacy of MRI in prenatal PA was higher, with sensitivity and accuracy not lower than that of US examination, and it was highly consistent with intraoperative or case confirmation. On the other hand, in the judgment of the type of PA, especially in the diagnosis of PA, the difference between MRI and US examinations was statistically huge. In comparison of the other two types of PA, the difference between MRI and US examinations was not statistically marked, but the detection rate of MRI examination was higher than the rate of US examination. This was because when implantable PA occurred, the placenta passed through the decidua basalis and entered the myometrium. The boundary between the placenta and the decidua basalis disappeared and the intact myometrium was interrupted. Besides, the degree of PA was between placental adhesion and penetration, which required stricter resolution and spatial resolution of soft tissue, and the clinical treatment of placental implantation was usually based on the degree of PA. The deeper the degree of implantation, the more cases of adverse reactions would occur in pregnant women. Therefore, it was of great significance to have a certain judgment on the situation of PA before surgery. The results of this study disclosed that the efficiency of MRI in diagnosing PA was better than that of US, and the difference between the two examination techniques was statistically substantial. Compared with US, the efficiency of MRI in diagnosing PA was not too obvious. However, MRI examination can provide more reliable image information for clinical diagnosis and treatment of PA in terms of implant type, which can greatly reduce the delay of optimal treatment time due to unpredictable conditions or blind treatment by obstetricians that endangers the life and health of the maternal fetus.

## 5. Conclusion

In this study, the MRI detection model was established based on IPSO. The research results indicated that the MRI image based on IPSO was clearer and had higher sensitivity and specificity for the diagnosis of PA during pregnancy, especially for implantable PA and penetrated PA. Therefore, prenatal MRI can make timely and accurate diagnosis of PA during pregnancy, and it is of great significance to formulate a reasonable treatment plan for the clinic as soon as possible to improve the pregnancy outcome. The research results in this study have certain limitations due to the small sample size, and their reliability needs to be further expanded in the future research to confirm the sample size. It is also believed that with the continuous advancement of science and technology, medical imaging and computer-related technology will be more perfect for the diagnosis and treatment of PA, which will benefit the broad masses.

## Data Availability

The data used to support the findings of this study are available from the corresponding author upon request.

## References

[1] Chen Y., Wang L., Bao J. (2021). Persistent hypoxia induced autophagy leading to invasiveness of trophoblasts in placenta accreta. *Journal of Maternal-Fetal and Neonatal Medicine*.

[2] Bouvier A., Sentilhes L., Thouveny F. (2012). Planned caesarean in the interventional radiology cath lab to enable immediate uterine artery embolization for the conservative treatment of placenta accreta. *Clinical Radiology*.

[3] Doulaveris G., Ryken K., Papathomas D. (2020). Early prediction of placenta accreta spectrum in women with prior cesarean delivery using transvaginal ultrasound at 11 to 14 weeks. *American Journal of Obstetrics & Gynecology MFM*.

[4] Ganske A., Kolbe A. B., Thomas K., Hull N. (2021). Pediatric scurvy MRI appearance. *Radiology Case Reports*.

[5] Meller C. H., Garcia-Monaco R. D., Izbizky G. (2019). Non-conservative management of placenta accreta spectrum in the hybrid operating room: a retrospective cohort study. *CardioVascular and Interventional Radiology*.

[6] Sellmyer M. A., Desser T. S., Maturen K. E., Jeffrey R. B., Kamaya A. (2013). Physiologic, histologic, and imaging features of retained products of conception. *RadioGraphics*.

[7] Wu Y., Hatipoglu S., Alonso-Álvarez D. (2021). Fast and automated segmentation for the three-directional multi-slice cine myocardial velocity mapping. *Diagnostics*.

[8] Yang G., Chen J., Gao Z. (2020). Simultaneous left atrium anatomy and scar segmentations via deep learning in multiview information with attention. *Future Generation Computer Systems*.

[9] Li M., Wang C., Zhang H., Yang G., RAN M. V. (2020). MV-RAN: multiview recurrent aggregation network for echocardiographic sequences segmentation and full cardiac cycle analysis. *Computers in Biology and Medicine*.

[10] Knill C. N., Crandall R. S., Jurus D. T. (2021). Insufficient lactation leading to postpartum diagnosis of placenta accreta spectrum disorder in a primigravid patient. *Obstetrics and Gynecology*.

[11] Jauniaux E., Alfirevic Z., Bhide A. (2019). Placenta praevia and placenta accreta: diagnosis and management. *BJOG: An International Journal of Obstetrics and Gynaecology*.

[12] Silver R. M., Barbour K. D. (2015). Placenta accreta spectrum. *Obstetrics & Gynecology Clinics of North America*.

[13] Chen L., Shi H. F., Jiang H (2021). Correlation of an ultrasonic scoring system and intraoperative blood loss in placenta accreta spectrum disorders: a retrospective cohort study. *Biomedical and Environmental Sciences*.

[14] Lo T. K., Mok S. L., So C. H., Cheng L. F. (2021). Efficacy of targeted screening and proactive management for placenta accreta spectrum disorder in routine clinical settings. *International Journal of Gynecology and Obstetrics*.

[15] Matsubara S. (2021). Placenta accreta spectrum: “placenta accreta” is still used. *Archives of Gynecology and Obstetrics*.

[16] Aughwane R., Ingram E., Johnstone E. D., Salomon L. J., David A. L., Melbourne A. (2020). Placental MRI and its application to fetal intervention. *Prenatal Diagnosis*.

[17] Jin Y., Yang G., Fang Y. (2021). 3D PBV-Net: an automated prostate MRI data segmentation method. *Computers in Biology and Medicine*.

